# Comparison of dental treatment performed under general anesthesia between healthy children and children with special health care needs in a hospital setting, Saudi Arabia

**DOI:** 10.4317/jced.55060

**Published:** 2018-10-01

**Authors:** Shahed Al-Ogayyel, Sanaa Al-Haj Ali

**Affiliations:** 1 Dental intern, college of dentistry, Qassim University; 2 Associate professor in pediatric dentistry, department of orthodontics and pediatric dentistry, college of dentistry, Qassim University, Qassim, Kingdom of Saudi Arabia

## Abstract

**Background:**

The aim of this retrospective study was to assess and compare the dental treatments performed under general anesthesia (GA) between healthy children and children with special health care needs (SHCN) according to age group and gender at king Fahd hospital, Dhahran, Saudi Arabia.

**Material and Methods:**

Data was retrieved from the records of 304 healthy and SHCN children 1 18 years of age who received dental rehabilitation under GA in the period 2015-2018. The dental treatment modalities were compared in the two groups and differences according to age group and gender were reported.

**Results:**

Compared to healthy children, children with SHCN received significantly less pulp therapy treated teeth, and restored primary teeth. While, they received significantly more extracted teeth, and restored permanent teeth (*P*<0.05). In both groups, younger children (≤ 6 years) received significantly more crowns, pulp therapy treated teeth, and restored primary teeth than older children (> 6 years) (*P*<0.05). While, they received significantly less fissure sealed teeth, extracted primary teeth, and restored permanent teeth (*P*<0.05). No gender difference was found among children with SHCN; however, healthy boys ≤ 6 years received significantly more extracted primary teeth than girls of same age group, while healthy boys > 6 years received significantly more pulp therapy treated teeth than girls of same age group (*P*<0.05).

**Conclusions:**

Healthy children had different approaches for treatment under GA than children with SHCN. The use of radical treatment approaches like extraction in children with SHCN and the lessened preference toward pulp therapy coupled with greater need for permanent teeth restorations when compared to healthy children were observed. Greater emphasis on oral health education and preventive strategies for children with SHCN is required. It is important to educate their parents/caregivers on the importance of establishing early dental home.

** Key words:**Dental treatment, general anesthesia, special health care needs.

## Introduction

Behavioral management has a crucial role in facilitating the provision of high quality dental treatment for children and ensuring safe and pain free environment (1). While some children receive treatment in a dental chair, for more severe cases, SHCN patients or child behavioral problems GA may be required (2). The American academy of pediatric dentistry defines SHCN as “any physical, developmental, mental, sensory, behavioral, cognitive, or emotional impairment or limiting condition that requires medical management, health care intervention, and/or use of specialized services or programs. The condition may be developmental or acquired and may cause limitations in performing daily self-maintenance activities or substantial limitations in a major life activity” (3). Children with SHCN are prone to have poor oral hygiene and poorer periodontal status with untreated caries teeth, (4-6) since most of them have limited motor and sensory coordination and are consequently unable to care for themselves and must rely on their parents or caregivers for general care (7). In addition, these patients frequently show high anxiety levels, a low level of cooperation, and mood swings, which form a barrier to dental treatment in the dental chair (1). Studies on several populations have shown high unmet dental needs among children with SHCN (7-10). Therefore, GA presents a very important option for dentists to perform comprehensive management of children with SHCN for the above mentioned reasons and to overcome the potential risks, excessive stress, and inability to offer high-quality dental treatment in the dental chair (11).

Several studies compared the dental treatments performed under GA between children with SHCN and healthy children (12-17). The majority of these studies reported higher frequency of extractions in children with SHCN when compared to healthy children (13-17). Some of these studies reported significantly less frequency of pulp therapy and stainless steel crowns among children with SHCN (12,15,16), while others reported significantly more restorative treatment among this special group of children (13,14).

Little emphasis is made on differences in dental treatment performed under GA between healthy children and children with SHCN in the Arab region in general and Saudi Arabia in particular. Therefore, the aim of this study was to assess and compare the dental treatments performed under GA between healthy children and children with SHCN according to age group and gender at king Fahd hospital, Dhahran, Saudi Arabia.

## Material and Methods

-Subjects

In this study, the retrospective complete records of patients 1‑18 years of age who received dental rehabilitation under GA in the pediatric dental department of king Fahd hospital, Dhahran, Saudi Arabia from January 2015 to January 2018 were evaluated. The patients were divided into two groups; those with SHCN who had at least one type of mental or physical disability were assigned to the SHCN group and those with neither mental nor physical disabilities who had difficulty cooperating were included in the healthy group. Further subgrouping was done to the patients according to the age group (≤ 6 years or >6 years) and gender (boys/girls). This study was approved by Qassim University college of dentistry ethical committee (reference number: EA/5005/2017).

-Data collection

The procedures observed from the patient treatment records included the total number of restored primary teeth, restored permanent teeth (including those which were treated with preventive resin restoration), the total number of crowns (including stainless steel crowns (SSCs), composite strip crowns, zirconia, and pre-veneered SSCs), the total number of fissure sealed primary and permanent teeth, the total number of pulp treated primary and permanent teeth (including indirect pulp treatment, direct pulp treatment, pulpotomy, pulpectomy, and root canal treatment procedures), the total number of extracted primary teeth, and extracted permanent teeth (including supernumerary teeth extraction).

All patients who were included in this study had appropriate dental and anesthetic pre-operative assessments. Dental assessment included a dental and medical history, clinical examination, oral radiographs and appropriate hematological tests. All dental treatments were carried out under GA with naso-endotracheal intubation by a senior pediatric dentist and in case of uneventful procedure patients were discharged 2 hours after recovery.

-Statistical analyses

Data were recorded and analyzed using three-way ANOVA and Bonferroni post hoc test with *p* < 0.05 indicating significance using the SPSS computer software (SPSS Version 20, Chicago, IL, USA).

## Results

A total of 304 children were included in this study; the boys (134/304; 44.1 %) to girls (170/304; 55.9 %) ratio in our study population was 1:1.3. The ratio of healthy children (220/304; 72.4%) to children with SHCN (84/304; 27.6%) was 2.6:1. While, the ratio of children ≤ 6 years of age (211/304; 69.4%) to those > 6 years of age (93/304; 30.6%) was 2.3:1. Out of 220 healthy children, 164 children (74.5%) were ≤ 6 years of age, while 47 SHCN children out of 84 (56%) were from the younger age group (≤ 6). About 61.4% (135/220) of healthy children who received treatment were girls compared to 41.7% girls (35/84) of treated children with SHCN. The two major underlying problems among children with SHCN were autism (50%) and cerebral palsy (30%).

**Figure 1:**
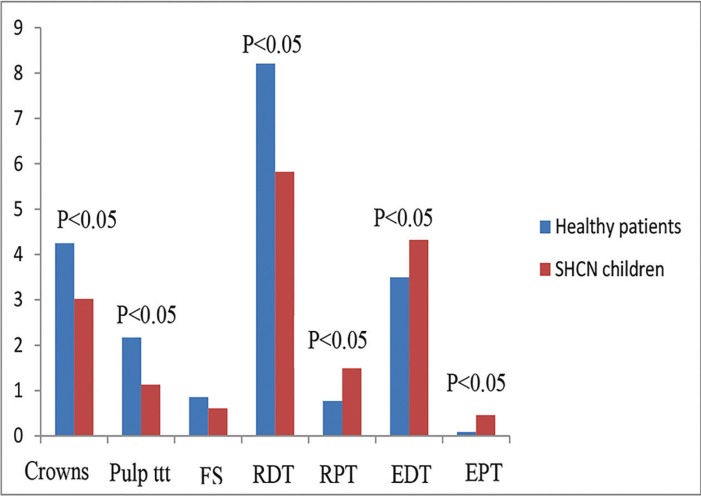
Dental treatment modalities performed in healthy children and children with special health care needs. Pulp ttt: pulp treatment, FS: fissure sealant, RDT: restored deciduous teeth, RPT: restored permanent teeth, EDT: extracted deciduous teeth, EPT: extracted permanent teeth.

 Table 1 shows the treatment modalities and mean number of teeth treated in healthy children and children with SHCN according to age group (≤6 years and >6 years) and in total. Compared to healthy children, children with SHCN had significantly less mean number of pulp therapy treated teeth, and restored primary teeth (*P*=0.001). On the other hand, they had significantly more mean number of extracted primary teeth and permanent teeth (*P*=0.015). In addition, children with SHCN had significantly greater mean number of restored permanent teeth than healthy children (*P*=0.005) (Fig. 1).

**Table 1:**
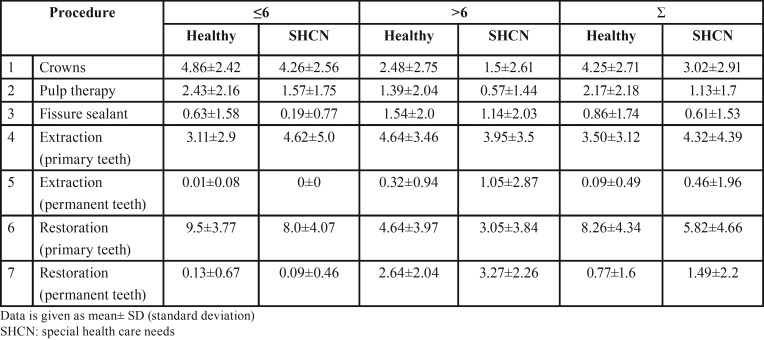
The dental treatment modalities performed in healthy children and children with special health care needs according to age group (≤6 years and >6 years) and in total.

**Figure 2:**
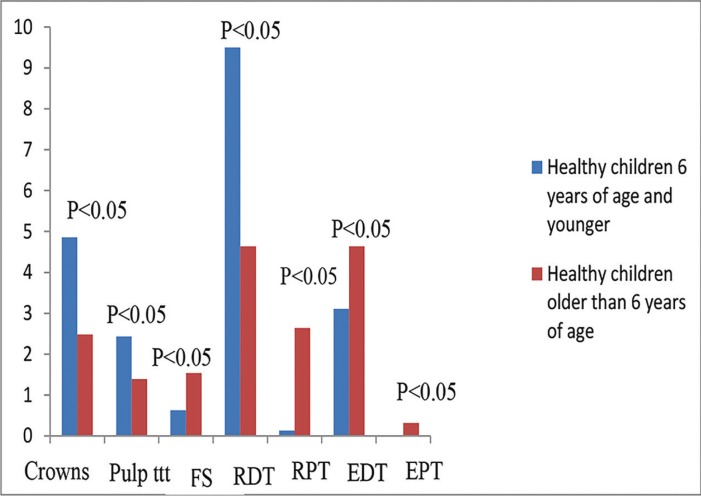
Dental treatment modalities performed in healthy children according to age group. Pulp ttt: pulp treatment, FS: fissure sealant, RDT: restored deciduous teeth, RPT: restored permanent teeth, EDT: extracted deciduous teeth, EPT: extracted permanent teeth.

**Figure 3:**
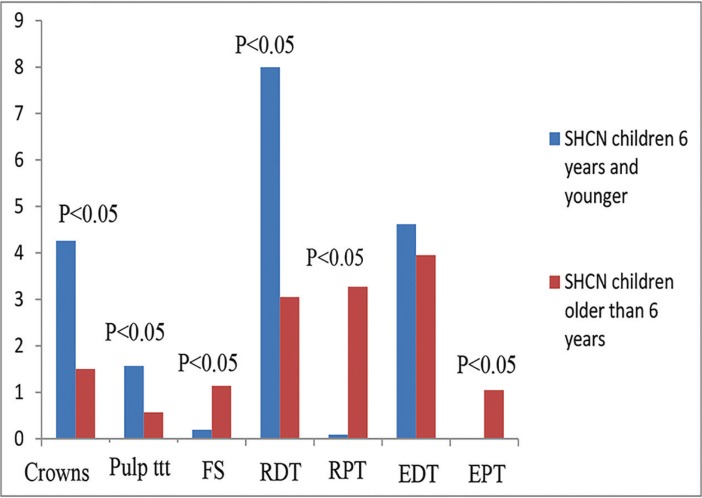
Dental treatment modalities performed in children with special health care needs according to age group. Pulp ttt: pulp treatment, FS: fissure sealant, RDT: restored deciduous teeth, RPT: restored permanent teeth, EDT: extracted deciduous teeth, EPT: extracted permanent teeth.

When both age groups were compared together, healthy children who were ≤ 6 years of age received significantly greater mean number of crowns (*P*< 0.001), pulp therapy treated teeth (*P*<0.001), and restored primary teeth than those who were> 6 years of age (*P*<0.001). On the other hand, they received significantly lower mean number of fissure sealed teeth (*P*=0.027), extracted primary teeth (*P*=0.015) and permanent teeth (P=0.035), and restored permanent teeth (*P*< 0.001) (Fig. 2). On the contrary, children with SHCN who were ≤ 6 years of age received significantly greater mean number of crowns (*P*=0.012), pulp therapy treated teeth, and restored primary teeth than those who were > 6 years of age (*P*=0.01 and <0.001 respectively). While, they received significantly lower mean number of fissure sealed teeth (*P*=0.027), extracted and restored permanent teeth than those who were > 6 years of age (*P*<0.001) (Fig. 3).

 Table 2 and  Table 3 show the treatment modalities and mean number of teeth treated in healthy children and children with SHCN according to gender and age group; those who were ≤ 6 years of age in  Table 2 and those who were > 6 years of age in  Table 3. Healthy boys who were ≤ 6 years of age received significantly greater mean number of extracted primary teeth than healthy girls from the same age group, while those who were > 6 years of age received significantly greater mean number of pulp therapy treated teeth than girls > 6 years of age. The rest of the differences were insignificant (*P*>0.05).

**Table 2:**
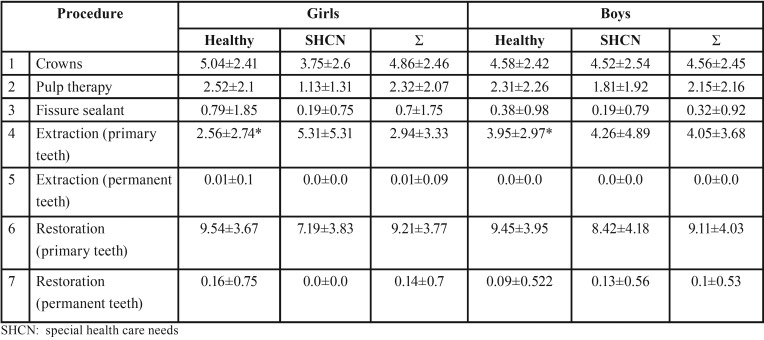
The dental treatment modalities performed in healthy children and children with special health care needs ≤6 years of age according to gender. * statistically significant difference.

**Table 3:**
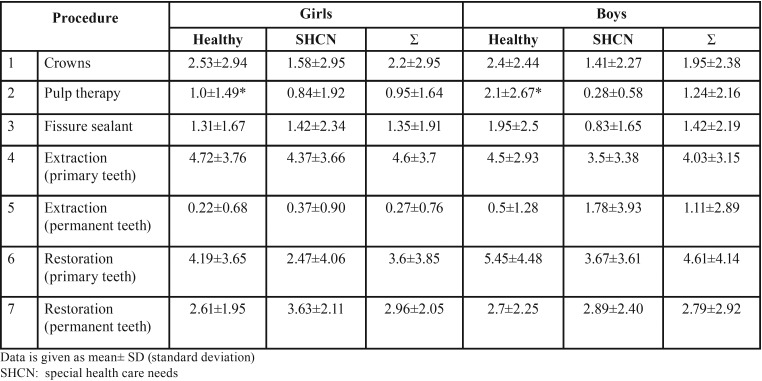
The dental treatment modalities performed in healthy children and children with special health care needs > 6 years of age according to gender. * statistically significant difference.

## Discussion

SHCN patients beyond the age of majority are commonly seen by pediatric dentists and they constitute an integral part of the specialty of pediatric dentistry. Dental treatment for these patients is commonly performed under GA in hospital-based setting as it is the ideal location to ensure safety of these patients (18). In this study, children with SHCN constituted about one third of children treated under GA. Similar percentage (23%) was reported in another study in Saudi Arabia (19). In addition, more children with SHCN were from the older age group category (>6 years) at the time of treatment when compared to healthy children (44% vs 25.5% for healthy children); a finding which was also reported in previous studies (16,19). The two major underlying problems in children with SHCN who were treated under GA in this study were autism and cerebral palsy. These two conditions were also reported by de Nova García *et al.* (20) as being the most common diagnosis for SHCN children treated under GA.

In this study, primary teeth restorations followed by primary teeth extractions were the two most frequently performed procedures under GA for children with SHCN. Similar finding was reported by Al-Malik and Al-Sarheed (19) and Ibricevic *et al.* (12). However, when the treatment modalities between healthy children and children with SHCN were compared, more primary and permanent teeth extractions and permanent teeth restorations and less primary and permanent pulp therapy treated teeth and primary teeth restorations were performed for children with SHCN when compared to healthy children with statistically significant differences. Similar findings were reported in previous studies (15-17). This may indicate that at the time GA was performed, children with SHCN presented with higher caries activity in both dentitions (primary and permanent) coupled with more un-restorable primary teeth when compared to healthy children, consequently the decision was in favor of extraction for the un-restorable teeth in these patients and restoration of teeth with limited caries activity. On the other hand, when the carious activity was extensive with potential or actual damage to the pulp, the decision seemed to favor extraction over some form of pulp therapy. This interpretation needs to be taken with caution, however, as no assessment was made in this study for the decayed missing and filled primary teeth (dmf‑t) and permanent teeth (DMF‑T) scores in both groups studied which can be considered a limitation. Nevertheless, Brown (21) reported higher mean dmft score for 5 year- old medically compromised patients in Riyadh, Saudi Arabia when compared to healthy children (9.91 vs. 6.25), while that was not a population-based study as well, it is very likely that this pattern reflects that of the community at large as many overseas studies also found that medically compromised children had a high mean dmft even when compared to healthy children (13,22,23).

Another explanation of our findings would be that the treatment decision got influenced by the health status of the child. This observation was also suggested in previous studies (12,16,17,24). Dentists may prefer a less complex and more radical dental procedure under GA for a SHCN child to avoid complications or the necessity for retreatment than a healthy child (16,24). This makes sense even for healthy children in cases of frank, invasive periapical pathology. In that case extraction rather than heroic pulpal modalities and esthetic crowns should be done as definitive treatment, as intervention intended to minimize further complications or follow-ups should be the approach to take when working under GA with high risk patients, just as full coverage is the order of the day rather than “fillings” for grossly decayed hard tissue (25).

In this study, regardless of the medical health of the child (healthy or SHCN), younger children (≤ 6 years) received more crowns, pulp therapy treated teeth, and primary teeth restorations while they received less fissure sealed teeth, extracted primary and permanent teeth than older children (> 6 years) with statistically significant differences. This finding is in part in agreement with Chen *et al.* (26) who found less mean number of crowns and pulpotomized teeth in children >6 years and higher mean number of extracted permanent teeth than younger children. This would be likely due to the fact that most primary teeth among this age group were at a late stage and would be exfoliated in a few years. In such circumstances, the need for pulp therapy and crowns may have been converted to tooth extraction.

When the influence of gender was assessed in this study, differences were only reported among healthy children where boys ≤ 6 years of age had significantly more primary teeth extractions than girls from the same age group while those > 6 years of age had significantly more pulp therapy treated teeth. Dhahran is a major city in the eastern region of Saudi Arabia. Previous caries prevalence studies among children from other cities located in the eastern region of Saudi Arabia failed to demonstrate any gender difference (27,28). However, the findings from this study suggest that there might be gender difference in Dhahran, as more primary teeth extractions among boys of the younger age group category may indicate higher caries activity when compared to girls of this age group. Consequently, we strongly encourage further research in this area.

In summary, the findings from this study reveal an overall difference in the treatment approach under GA between healthy children and children with SHCN. The use of more radical treatment approaches like extraction in children with SHCN and the lessened preference toward pulp therapy coupled with greater need for permanent teeth restorations when compared to healthy children were observed. Therefore, more emphasis on oral health education and preventive strategies for children with SHCN is required. Education of parents/caregivers is critical for ensuring appropriate and regular supervision of daily oral hygiene as majority of children with SHCN have limited ability to perform daily oral hygiene measures consequently parents/caregivers should provide the appropriate oral care when the patient is unable to do so adequately. Caregivers’ lack of awareness and knowledge can also limit a SHCN child from seeking preventive dental care especially when the relationship between oral health and general health is not well understood. As the dental home provides an opportunity to implement individualized preventive oral health practices and reduces the patient’s risk of preventable dental/oral disease. It is extremely important to educate the parents/caregivers of children with SHCN on the importance of establishing early dental home.
